# Robust stability analysis of impulsive complex-valued neural networks with time delays and parameter uncertainties

**DOI:** 10.1186/s13660-017-1490-0

**Published:** 2017-09-11

**Authors:** Yuanshun Tan, Sanyi Tang, Jin Yang, Zijian Liu

**Affiliations:** 1 College of Mathematics and Statistics, Chongqing Jiaotong University, Chongqing, 400074 China; 2 College of Mathematics and Statistics, Shaanxi Normal University, Shanxi, 710062 China

**Keywords:** complex-valued neural networks, robust stability, delay, impulsive

## Abstract

The present study considers the robust stability for impulsive complex-valued neural networks (CVNNs) with discrete time delays. By applying the homeomorphic mapping theorem and some inequalities in a complex domain, some sufficient conditions are obtained to prove the existence and uniqueness of the equilibrium for the CVNNs. By constructing appropriate Lyapunov-Krasovskii functionals and employing the complex-valued matrix inequality skills, the study finds the conditions to guarantee its global robust stability. A numerical simulation illustrates the correctness of the proposed theoretical results.

## Introduction

Robustness is the ability of maintaining the performance of the controlling system under certain parameter perturbations. When these inner structural disturbances result in the instability of the system, additional control mechanisms should be used to improve these flimsy properties. When the control method is introduced, the uncertainty and error brought by the control itself also become another disturbance factor of the system. In real life and engineering practices, perturbations of system characteristics or parameters are often unavoidable. Perturbations exist for two main reasons. One is that the actual measurement is not accurate and usually deviates from its designed value. The other is slow drift of characteristics or parameters, which is influenced by the environmental factors in the running process of the system. When these uncertainties or random disturbances exist, the questions how and in what range to control the quality of the system or maintain its characteristics are of great importance. Therefore, robustness has become an important research topic in control theory, and it is also a basic problem that must be considered in the design of almost all kinds of control systems, such as image and signal processing, combinatorial optimization problems, pattern recognition, etc. It has attracted great attention of the scholars that work with neural networks [1–6].

For many applications of neural networks, on the one hand, the states change rapidly at a fixed time, and the duration of these abrupt changes is often neglected, assuming that they are caused by jumps. Such processes are studied by impulsive differential equations (for the relative theorems, we refer to [7]), and there are numerical applications of such equations in science and technology, mass services, etc. [8–11]. On the other hand, due to the neural processing and signal transmission, a time delay often occurs, which may cause instability and a poor performance of the system [12]. Generally, delays may be caused by the measuring process and therefore the effect of time delay is common. Also many efforts are being made as regards the delay-dependent stability analysis of neural networks [13–23].

In the application of neural networks, complex signals are usually preferable [24–29], so it is necessary to analyze complex-valued neural networks (CVNNs), which deal with the complex-valued date, weight and neuron activation functions. However, much work mainly focuses on the boundedness, *μ*-stability, power-stability, exponent-stability, etc. [3, 18, 30–32], and little work considers the robust stability of neural networks with time delay and impulse in the complex domain. In [2, 6], the authors investigated a kind of recurrent CVNNs with time delays, but the activation functions are separated by real and imaginary parts and the analysis methods are also similar to those in their real domain. Therefore, the stability criteria cannot be applied if the activation functions cannot be expressed by separating their real and imaginary parts or if they are unbounded. Moreover, compared to real-valued neural networks, the advantage of CVNNs is that they can directly deal with two-dimensional data, which can also be processed by real-valued neural networks but then require double neurons. Consequently, as a class of complex-valued systems, CVNNs have undergone a growing number of studies that explore the application of neural networks. Therefore, the present study considers the robust stability of CVNNs with discrete time delay and impulse, which is valid regardless of whether the activation functions are separated or not. The relative results are extension of those in reference [2, 6]. Therefore, the robust stability for impulsive CVNNs with time delays is considered in this paper. Furthermore, compared with [2] and [6], the robust stability criteria for the addressed neural networks are valid regardless of whether the activation functions can be separated or not.

The structure of this paper is arranged as follows. Section 2 gives some preliminaries, including some notations and important lemmas, introducing the complex-valued recurrent networks model. The existence and uniqueness of the equilibrium are proved by using the homeomorphism mapping principle in Section 3. In Section 4, the global robust stability of the neural networks is investigated by building the proper Lyapunov functions. An example is given to illustrate the correction of our results. In Section 5, we conclude the paper.

## Problems formulation and preliminaries

Some notations of this paper are presented here firstly. *i* denotes the imaginary unit, *i.e.*, $i=\sqrt{-1}$. $\mathbb{C}^{n}$, $\mathbb{R}^{m\times n}$, and $\mathbb{C}^{m\times n}$ represent the set of *n*-dimensional complex vectors, $m\times n$ real matrices, and complex matrices, respectively. The subscripts *T* and ∗ denote matrix transposition and matrix conjugate transposition, respectively. For complex vector $z\in\mathbb{C}^{n}$, let $|z|=(|z_{1}|,|z_{2}|,\ldots ,|z_{n}|)^{T}$ be the module of the vector *z* and $\|z\|=\sqrt{\sum_{i=1}^{n}|z_{i}|^{2}}$ be the norm of the vector *z*. For complex matrix $A=(a_{ij})_{n\times n}\in\mathbb{C}^{n\times n}$, let $|A|=(|a_{ij}|)_{n\times n}$ denote the module of the matrix *A* and $\|A\|=\sqrt{\sum_{i=1}^{n}\sum_{j=1}^{n} |a_{ij}|^{2}}$ denote the norm of the matrix *A*. *I* denotes the identity matrix with appropriate dimensions. The notation $X\geq Y$ (or $X>Y$) means that $X-Y$ is positive semi-definite (or positive definite). In addition, $\lambda_{\max}(P)$ and $\lambda_{\min}(P)$ are defined as the largest and the smallest eigenvalue of positive definite matrix *P*, respectively.

Motivated by [2], we consider the following impulsive CVNN model with time delays:

1 $$ \textstyle\begin{cases} \dot{z}_{i}(t)=-c_{i} z_{i}(t)+\sum_{j=1}^{n} a_{ij}f_{j}(z_{j}(t)) \\ \phantom{\dot{z}_{i}(t)=}{}+\sum_{j=1}^{n} b_{ij} f_{j}(z_{j}(t-\tau_{j}))+J_{i}, & t\geq 0, t\neq t_{k},\\ \Delta z_{i}(t_{k})= I_{ik}(z_{i}(t_{k}^{-})), & k=1,2,\ldots, i=1,2,\ldots,n, \end{cases} $$

where *n* is the number of neurons, $z_{i}(t)\in\mathbb{C}$ denotes the state of neuron *i* at time *t*, $f_{j}(t)$ is the complex-valued activation function, $\tau_{j}$ ($j=1,2,\ldots,n$) are constant time delays and satisfy $0\leq \tau_{j}\leq\rho$, $c_{i}\in\mathbb{R}$ with $c_{i} > 0$ is the self-feedback connection weight, $a_{ij}\in\mathbb{C}$ and $b_{ij}\in\mathbb{C}$ are the connection weights, and $J_{i}\in\mathbb{C}$ is the external input. Here $I_{ik}$ is a linear map, $\Delta z_{i}(t_{k})=z_{i}(t_{k}^{+})-z_{i}(t_{k}^{-})$ is the jump of $z_{i}$ at moments $t_{k}$, and $0< t_{1}< t_{2}<\cdots$ is a strictly increasing sequence such that $\lim_{k\to\infty}t_{k}=+\infty$.

We rewrite (1) in the equivalent matrix-vector form

2 $$ \textstyle\begin{cases} \dot{z}(t)=-C z(t)+A f(z(t))+B f(z(t-\tau))+J,\\ \Delta z(t_{k})=I(z(t_{k}^{-})),\quad k=1,2,\ldots, \end{cases} $$

where $z(t)=(z_{1}(t),z_{2}(t),\ldots,z_{n}(t))^{T}\in\mathbb{C}^{n}$, $C=\operatorname {diag}(c_{1},c_{2},\ldots,c_{n})$, $A=(a_{ij})_{n\times n}\in\mathbb{C}^{n\times n}$, $B=(b_{ij})_{n\times n}\in\mathbb{C}^{n\times n}$, $f(z(t))=(f_{1}(z_{1}(t)),f_{2}(z_{2}(t)),\ldots,f_{n}(z_{n}(t)))^{T}$, $f(z(t-\tau))=(f_{1}(z_{1}(t-\tau_{1})),f_{2}(z_{2}(t-\tau_{2})),\ldots ,f_{n}(z_{n}(t-\tau_{n})))^{T}$, $J=(J_{1},J_{2},\ldots,J_{n})^{T}\in\mathbb{C}^{n}$, $\Delta z(t_{k})=(\Delta z_{1}(t_{k}), \Delta z_{2}(t_{k}),\ldots,\Delta z_{n}(t_{k}))^{T}$, and $I(z(t_{k}^{-}))=(I_{1k}(z(t_{k}^{-})), I_{2k}(z(t_{k}^{-})),\ldots ,I_{nk}(z(t_{k}^{-})))^{T}$.

Assume that system (1) or (2) is supplemented with the initial values given by

3 $$ z_{i}(s)=\varphi_{i}(s), \quad s\in[- \rho,0], i=1,2,\ldots, $$

or in the equivalent vector form

4 $$ z(s)=\varphi(s), \quad s\in[-\rho,0], $$

where $\varphi_{i}(\cdot)$ is a complex-valued continuous function defined on $[-\rho, 0]$ and $\varphi(s)=(\varphi_{1}(s),\varphi_{2}(s),\ldots,\varphi_{n}(s))^{T}\in C([-\rho,0],\mathbb{C}^{n})$ with the norm $\|\varphi(s)\|=\sup_{s\in[-\rho,0]}\sqrt{\sum_{i=1}^{n}|\varphi_{i}(t)|^{2}}$.

The following assumptions will be needed in the study:

(**H1**) The parameters $C=\operatorname {diag}(c_{1}, c_{2}, \ldots, c_{n})$, $A=(a_{ij})_{n\times n}$, $B=(b_{ij})_{n\times n}$, and $J=(J_{1},J_{2},\ldots, J_{n})^{T}$ in neural system (1) are assumed to be norm-bounded and satisfy

$$\begin{aligned}& C_{I}=[\underline{C},\overline{C}]= \bigl\{ C=\operatorname {diag}(c_{1}, c_{2}, \ldots, c_{n}): 0< \underline{c}_{i}\leq c_{i}\leq\overline{c}_{i}, i=1,2,\ldots,n \bigr\} , \\& A_{I}=[\underline{A},\overline{A}]= \bigl\{ A=(a_{ij})_{n\times n}: \underline {a}^{R}_{ij}\leq a^{R}_{ij} \leq\overline{a}^{R}_{ij}, \underline{a}^{I}_{ij} \leq a^{I}_{ij}\leq\overline{a}^{I}_{ij}, i,j=1,2,\ldots,n \bigr\} , \\& B_{I}=[\underline{B},\overline{B}]= \bigl\{ B=(b_{ij})_{n\times n}: \underline {b}^{R}_{ij}\leq b^{R}_{ij} \leq\overline{b}^{R}_{ij}, \underline{b}^{I}_{ij} \leq b^{I}_{ij}\leq\overline{b}^{I}_{ij}, i,j=1,2,\ldots,n \bigr\} , \\& J_{I}=[\underline{J},\overline{J}]= \bigl\{ J=(J_{1},J_{2}, \ldots,J_{n})^{T}: \underline{J}_{i}^{R} \leq J_{i}^{R}\leq\overline{J}_{i}^{R}, \underline{J}^{I}_{i}\leq J_{i}^{I}\leq \overline{J}_{i}^{I},i=1,2,\ldots,n \bigr\} , \end{aligned}$$

where $a_{ij}=a^{R}_{ij}+\mathrm {i}a^{I}_{ij}$, $b_{ij}=b^{R}_{ij}+\mathrm {i}b^{I}_{ij}$, $J_{i}=J_{i}^{R}+\mathrm {i}J_{i}^{I}$, $\underline{C}=\operatorname {diag}(\underline{c}_{1}, \underline{c}_{2}, \ldots, \underline{c}_{n})$, $\overline{C}=\operatorname {diag}(\overline{c}_{1}, \overline{c}_{2}, \ldots, \overline{c}_{n})$, $\underline{A}=(\underline{a}_{ij})_{n\times n}$, $\overline{A}=(\overline{a}_{ij})_{n\times n}$, $\underline{B}=(\underline{b}_{ij})_{n\times n}$, $\overline{B}=(\overline{b}_{ij})_{n\times n}$, $\underline{J}=(\underline{J}_{1},\underline{J}_{2},\ldots,\underline{J}_{n})^{T}$, $\overline{J}=(\overline{J}_{1},\overline{J}_{2},\ldots,\overline{J}_{n})^{T}$ with $\underline{a}_{ij}=\underline{a}_{ij}^{R}+\mathrm {i}\underline{a}_{ij}^{I}$, $\overline{a}_{ij}=\overline{a}_{ij}^{R}+\mathrm {i}\overline{a}_{ij}^{I}$, $\underline{b}_{ij}=\underline{b}_{ij}^{R}+\mathrm {i}\underline{b}_{ij}^{I}$, $\overline{b}_{ij}=\overline{b}_{ij}^{R}+\mathrm {i}\overline{b}_{ij}^{I}$, $\underline{J}_{i}=\underline{J}_{i}^{R}+\mathrm {i}\underline{J}_{i}^{I}$, and $\overline{J}_{i}=\overline{J}_{i}^{R}+\mathrm {i}\overline{J}_{i}^{I}$.

(**H2**) For $i=1,2,\ldots,n$, the neuron activation function $f_{i}$ is continuous and satisfies

$$\bigl\vert f_{i}(z_{1})-f_{i}(z_{2}) \bigr\vert \leq\gamma_{i} \vert z_{1}-z_{2} \vert , $$

for any $z_{1},z_{2}\in\mathbb{C}$, where $\gamma_{i}$ is a real constant. Furthermore, define $\Gamma=\operatorname{diag}(\gamma_{1},\gamma_{2},\ldots,\gamma_{n})$.

### Definition 1

A function $z(t)\in C([\tau,+\infty),\mathbb{C}^{n})$ is a solution of system (1) satisfying the initial value condition (3), if the following conditions are satisfied:

(i) $z(t)$ is absolutely continuous on each interval $(t_{k}, t_{k+1})\subset[-\tau,+\infty)$, $k=1,2,\ldots$ ,
(ii) for any $t_{k}\in[0,+\infty)$, $k=1,2,\ldots$ , $z(t_{k}^{+})$ and $(z(t_{k}^{-}))$ exist and $z(t_{k}^{+})=z(t_{k})$.

### Definition 2

The neural network defined by (1) with the parameter ranges defined by (**H1**) is globally asymptotically robust stable if the unique equilibrium point $\check{z}=(\check{z}_{1},\check {z}_{2}, \ldots, \check{z}_{n})^{T}$ of the neural system (1) is globally asymptotically stable for all $C\in C_{I}$, $A\in A_{I}$, $B\in B_{I}$, and $J\in J_{I}$.

### Lemma 1

[10]

*For any* $a,b\in\mathbb{C}^{n}$, *if* $P\in\mathbb{C}^{n\times n}$ *is a positive definite Hermitian matrix*, *then* $a^{*}b+b^{*}a\leq a^{*}Pa+b^{*}P^{-1}b$.

### Lemma 2

See [10]

*A given Hermitian matrix*

$$S= \begin{pmatrix} S_{11} & S_{12} \\ S_{21} & S_{22} \end{pmatrix} < 0, $$

*where* $S_{11}^{*}=S_{11}$, $S_{12}^{*}=S_{21}$, *and* $S_{22}^{*}=S_{22}$, *is equivalent to any of the following conditions*:

(i) $S_{22}<0$ *and* $S_{11}-S_{12}S_{22}^{-1}S_{21}<0$,
(ii) $S_{11}<0$ *and* $S_{22}-S_{21}S_{11}^{-1}S_{12}<0$.

### Lemma 3

[10]

*If* $H(z):\mathbb{C}^{n}\to\mathbb{C}^{n}$ *is a continuous map and satisfies the following conditions*:

(i) $H(z)$ *is injective on* $\mathbb{C}^{n}$,
(ii) $\lim_{\|z\|\to\infty}\|H(z)\|=\infty$,

*then* $H(z)$ *is a homeomorphism of* $\mathbb{C}^{n}$ *onto itself*.

### Lemma 4

*Suppose* $A\in A_{I}$. *Let* *R* *and* *S* *be real positive diagonal matrices*. *The function* $f_{i}$ ($i=1,2,\ldots,n$) *satisfies* ($\mathbf{H2}$). *Then*, *for any* $z=(z_{1},z_{2},\ldots,z_{n})^{T}, \tilde{z}=(\tilde{z}_{1},\tilde{z}_{2},\ldots,\tilde{z}_{n})^{T}\in\mathbb {C}^{n}$, *the following inequalities hold*:

5 $$\begin{aligned}& z^{*}A^{*}Az\leq|z|^{*}\hat{A}^{*}\hat{A}|z|, \end{aligned}$$

6 $$\begin{aligned}& z^{*}RA^{*}SARz\leq|z|^{*}R\hat{A}^{*}S\hat{A}R|z|, \end{aligned}$$

7 $$\begin{aligned}& \bigl(f(z)-f(\tilde{z}) \bigr)^{*}A^{*}A \bigl(f(z)-f(\tilde{z}) \bigr) \leq|z-\tilde{z}|^{*}\Gamma \hat{A}^{*}\hat{A}\Gamma|z-\tilde{z}|, \end{aligned}$$

*where* $\hat{A}=(\hat{a}_{ij})_{n\times n}$, $\hat{a}_{ij}=\max\{|\underline{a}_{ij}|,|\overline{a}_{ij}|\}$, *and* $f(z)=(f_{1}(z_{1}),f_{2}(z_{2}),\ldots,f_{n}(z_{n}))^{T}$.

### Proof

It should be noted that $|a_{ij}|\leq\hat{a}_{ij}$ since $A\in A_{I}$. Then we calculate directly that

$$\begin{aligned} z^{*}A^{*}Az =& \sum_{i=1}^{n} \Biggl\vert \sum_{j=1}^{n} a_{ij}z_{j} \Biggr\vert ^{2} \\ \leq & \sum_{i=1}^{n} \Biggl(\sum _{j=1}^{n} \vert a_{ij} \vert \vert z_{j} \vert \Biggr)^{2} \\ \leq & \sum_{i=1}^{n} \Biggl(\sum _{j=1}^{n} \hat{a}_{ij} \vert z_{j} \vert \Biggr)^{2} \\ =& \vert z \vert ^{*}\hat{A}^{*}\hat{A} \vert z \vert . \end{aligned}$$

Hence inequality (5) holds.

Next we prove inequality (6). Let $S=\operatorname {diag}(s_{1},s_{2},\ldots ,s_{n})$ and $\tilde{S}=\operatorname {diag}(\sqrt{s}_{1},\sqrt{s}_{2},\ldots,\sqrt{s}_{n})$. Then $S=\tilde{S}^{2}$. It is obvious that $|Rz|=R|z|$ since *R* is a real positive diagonal matrix. From $A\in A_{I}$, it follows that $\underline{a}^{R}_{ij}\leq a^{R}_{ij}\leq\overline{a}^{R}_{ij}$ and $\underline{a}^{I}_{ij}\leq a^{I}_{ij}\leq\overline{a}^{I}_{ij}$ for all $i,j=1,2,\ldots,n$. Then $\sqrt{s_{i}}\underline{a}^{R}_{ij}\leq\sqrt{s_{i}}a^{R}_{ij}\leq\sqrt {s_{i}}\overline{a}^{R}_{ij}$ and $\sqrt{s_{i}}\underline{a}^{I}_{ij}\leq\sqrt{s_{i}}a^{I}_{ij}\leq\sqrt {s_{i}}\overline{a}^{I}_{ij}$, which means $\tilde{S}A \in\tilde{S}A_{I}$. Hence $\sqrt{s_{i}}\hat {a}_{ij}=\max\{|\sqrt{s_{i}}\underline{a}_{ij}|,|\sqrt{s_{i}}\overline {a}_{ij}|\}$. Noting that $\tilde{S}\hat{A}=(\sqrt{s_{i}}\hat{a}_{ij})_{n\times n}$, by inequality (5), we infer

$$\begin{aligned} z^{*}RA^{*}SARz =& (Rz)^{*}(\tilde{S}A)^{*}(\tilde{S}A) (Rz) \\ \leq & \vert Rz \vert ^{*}(\tilde{S}\hat{A})^{*}(\tilde{S}\hat{A}) \vert Rz \vert \\ = & \vert z \vert ^{*} R \hat{A}^{*} \tilde{S}\tilde{S}\hat{A}R \vert z \vert \\ =& \vert z \vert ^{*}R\hat{A}^{*}S\hat{A}R \vert z \vert . \end{aligned}$$

Therefore, inequality (6) holds.

Next we prove inequality (7). For simplicity, let $w_{i}=z_{i}-\tilde{z}_{i}$, $g_{i}=f_{i}(z_{i})-f_{i}(\tilde {z}_{i})$ ($i=1,2,\ldots,n$), $w=(w_{1},w_{2},\ldots,w_{n})^{T}$, and $g=(g_{1},g_{2},\ldots,g_{n})^{T}$. Then $|g_{i}|\leq\gamma_{i}|w_{i}|$ due to assumption (**H2**). So we calculate directly that

$$\begin{aligned} g^{*}A^{*}Ag =& \sum_{i=1}^{n} \Biggl\vert \sum_{j=1}^{n} a_{ij}g_{j} \Biggr\vert ^{2} \\ \leq & \sum_{i=1}^{n} \Biggl(\sum _{j=1}^{n} \vert a_{ij} \vert \vert g_{j} \vert \Biggr)^{2} \\ \leq & \sum_{i=1}^{n} \Biggl(\sum _{j=1}^{n} \gamma_{j}\hat{a}_{ij} \vert w_{j} \vert \Biggr)^{2} \\ =& \vert w \vert ^{*}\Gamma\hat{A}^{*}\hat{A}\Gamma \vert w \vert . \end{aligned}$$

Accordingly, inequality (7) holds. The proof is completed. □

## Existence and uniqueness of equilibrium point

In this section, we will give the sufficient conditions to prove the existence and uniqueness of equilibrium for system (1). An equilibrium solution of (1) is a constant complex vector $\check{z}\in\mathbb{C}^{n}$, which satisfies

8 $$ -C\check{z}+Af(\check{z})+Bf(\check{z})+J=0 $$

and $I_{k}(\check{z})=0$, $k=1,2,\ldots$ , when *ž* is the impulsive jump.

Hence, proving the existence and uniqueness of solution (8) is equivalent to proving the existence of a unique zero point of map $\mathcal{H}:\mathbb{C}^{n}\to\mathbb{C}^{n}$, where

9 $$ \mathcal{H}(z)=-Cz+Af(z)+Bf(z)+J. $$

We have the following theorem.

### Theorem 1

*For the CVNN defined by* (1), *assume that the network parameters and the activation function satisfy assumptions* $(\mathbf{H1})$ *and* $(\mathbf{H2})$, *respectively*. *Then the neural network* (1) *has a unique equilibrium point for every input vector* $J=(J_{1},J_{2},\ldots,J_{n})^{T}\in\mathbb{C}^{n}$, *if there exist two real positive diagonal matrices* *U* *and* *V* *such that the following linear matrix inequality* (*LMI*) *holds*:

10 $$ \begin{pmatrix} -2U\underline{C}+\Gamma V\Gamma& U(\hat{A}+\hat{B})\\ (\hat{A}^{*}+\hat{B}^{*})U & -V \end{pmatrix} < 0, $$

*where* $\hat{A}=(\hat{a}_{ij})_{n\times n}$, $\hat{B}=(\hat {b}_{ij})_{n\times n}$, $\hat{a}_{ij}=\max\{|\underline{a}_{ij}|,|\overline{a}_{ij}|\}$, *and* $\hat{b}_{ij}=\max\{|\underline{b}_{ij}|,|\overline{b}_{ij}|\}$.

### Proof

We will use the homeomorphism mapping theorem on the complex domain to prove the theorem, that is, to show the map $\mathcal{H}(z)$ is a homeomorphism of $\mathbb{C}^{n}$ onto itself.

First, we prove that $\mathcal{H}(z)$ is an injective map on $\mathbb{C}^{n}$. Let $z,\tilde{z}\in\mathbb{C}^{n}$ with $z\neq\tilde{z}$, such that $\mathcal{H}(z)=\mathcal{H}(\tilde{z})$. Then

11 $$ \mathcal{H}(z)-\mathcal{H}(\tilde{z})=-C(z-\tilde {z})+(A+B) \bigl(f(z)-f(\tilde{z}) \bigr)=0. $$

Multiplying both sides of (11) by $(z-\tilde{z})^{*}U$, we obtain

12 $$ 0= -(z-\tilde{z})^{*}U C(z-\tilde{z})+(z-\tilde{z})^{*}U(A+B) \bigl(f(z)-f(\tilde{z}) \bigr). $$

Then taking the conjugate transpose of (12) leads to

13 $$ 0= -(z-\tilde{z})^{*} C U^{*} (z-\tilde{z})+ \bigl(f(z)-f(\tilde {z}) \bigr)^{*} \bigl(A^{*}+B^{*} \bigr)U^{*}(z-\tilde{z}). $$

From (12), (13) and Lemmas 1 and 4, we have

14 $$\begin{aligned} 0 =& -(z-\tilde{z})^{*}(UC+ CU) (z-\tilde{z}) \\ &{}+(z-\tilde{z})^{*}U(A+B) \bigl(f(z)-f(\tilde{z}) \bigr) \\ &{}+ \bigl(f(z)-f(\tilde{z}) \bigr)^{*} \bigl(A^{*}+B^{*} \bigr)U(z-\tilde{z}) \\ \leq& -(z-\tilde{z})^{*}(U C+C U) (z-\tilde{z}) \\ &{}+(z-\tilde{z})^{*}U(A+B)V^{-1} \bigl(A^{*}+B^{*} \bigr)U(z-\tilde{z}) \\ &{}+ \bigl(f(z)-f(\tilde{z}) \bigr)^{*}V \bigl(f(z)-f(\tilde{z}) \bigr) \\ \leq & |z-\tilde{z}| \bigl[-2U\underline{C}+U(\hat{A}+\hat{B})V^{-1} \bigl(\hat {A}^{*}+\hat{B}^{*} \bigr)U \bigr]|z-\tilde{z}| \\ &{}+ \bigl(f(z)-f(\tilde{z}) \bigr)^{*}V \bigl(f(z)-f(\tilde{z}) \bigr). \end{aligned}$$

Since *V* is a positive diagonal matrix, from assumption (**H2**) we get

15 $$\begin{aligned} \bigl(f(z)-f(\tilde{z}) \bigr)^{*}V \bigl(f(z)-f(\tilde{z}) \bigr) \leq & (z- \tilde{z})^{*} \Gamma V\Gamma(z-\tilde{z}) \\ =& |z-\tilde{z}|^{*} \Gamma V\Gamma|z-\tilde{z}|. \end{aligned}$$

It follows from (14) and (15) that

16 $$ 0\leq|z-\tilde{z}|^{*} \Omega|z-\tilde{z}|, $$

where $\Omega=-2U\underline{C}+\Gamma V\Gamma+U(\hat{A}+\hat {B})V^{-1}(\hat{A}^{*}+\hat{B}^{*})U$. From Lemma 2 and the LMI (10), we know $\Omega < 0$. Then $z-\tilde{z}=0$ due to (16). Therefore, $\mathcal {H}(z)$ is an injective map on $\mathbb{C}^{n}$.

Secondly, we prove $\|\mathcal{H}(z)\|\to\infty$ as $\|z\| \to\infty$. Let $\widetilde{\mathcal{H}}(z)=\mathcal{H}(z)-\mathcal{H}(0)$. By Lemmas 1 and 4, we have

$$\begin{aligned}& z^{*}U\widetilde{\mathcal{H}}(z)+ \widetilde{\mathcal{H}}(z)^{*}Uz \\& \quad= -z^{*}(UC+CU)z +z^{*}U(A+B) \bigl(f(z)-f(0) \bigr) \\& \qquad{}+ \bigl(f(z)-f(0) \bigr)^{*} \bigl(A^{*}+B^{*} \bigr)Uz \\& \quad\leq-z^{*}(UC+CU)z \\& \qquad{}+z^{*}U(A+B)V^{-1} \bigl(B^{*}+C^{*} \bigr)Uz \\& \qquad {}+ \bigl(f(z)-f(0) \bigr)^{*}V \bigl(f(z)-f(0) \bigr) \\& \quad\leq|z| \bigl[-2U\underline{C}+U(\hat{A}+\hat{B})V^{-1} \bigl( \hat{A}^{*}+\hat {B}^{*} \bigr)U \bigr]|z| +|z|^{*}\Gamma V\Gamma|z| \\& \quad\leq|z|^{*} \Omega|z|\leq-\lambda_{\min}(-\Omega)\|z\|^{2}. \end{aligned}$$

An application of the Cauchy-Schwarz inequality yields

$$\lambda_{\min}(-\Omega)\|z\|^{2}\leq2 \bigl\Vert z^{*} \bigr\Vert \|U\| \bigl\Vert \widetilde {\mathcal{H}}(z) \bigr\Vert . $$

When $z\neq0$, we have

$$\bigl\Vert \widetilde{\mathcal{H}}(z) \bigr\Vert \geq\frac{\lambda_{\min}(-\Omega )\|z\|}{2 \|U\|}. $$

Therefore, $\|\widetilde{\mathcal{H}}(z)\|\to\infty$ as $\|z\| \to \infty$, which implies $\|\mathcal{H}(z)\|\to\infty$ as $\|z\| \to \infty$. We know that $\mathcal{H}(z)$ is a homeomorphism of $\mathbb{C}^{n}$ from Lemma 3, thus system (1) has a unique equilibrium point. □

## Global robust stability results

In this section, we will investigate the global robust stability of the unique equilibrium point for system (1). Firstly, the following assumption for the impulsive operators is needed: (**H3**) For $i=1,2,\ldots,n$ and $k=1,2,\ldots$ , $I_{ik}(\cdot)$ is such that

$$ I_{ik} \bigl(z_{i} \bigl(t_{k}^{-} \bigr) \bigr) = - \delta_{ik} \bigl(z_{i} \bigl(t_{k}^{-} \bigr)- \check{z}_{i} \bigr), $$

where $\delta_{ik}\in[0,2]$ is a real constant, and $\check{z}_{i}$ is the *i*th component of the equilibrium point $\check{z}=(\check {z}_{1},\check{z}_{2},\ldots,\check{z}_{n})^{T}$. Then we have the following global robust stability theorem.

### Theorem 2

*Suppose the conditions of Theorem* 1 *and* $(\mathbf{H3})$ *hold*. *The equilibrium point of system* (1) *is globally robust stable*, *if there exist two real positive diagonal matrices* $P=\operatorname{diag}(p_{1},p_{2},\ldots,p_{n})$ *and* $Q=\operatorname{diag}(q_{1},q_{2},\ldots,q_{n})$, *such that the following linear matrix inequalities hold*:

17 $$ \begin{pmatrix} -\underline{C}P+\Gamma\hat{A}^{*}\hat{A}\Gamma& P\\ P & -I \end{pmatrix} < 0 $$

*and*

18 $$ \begin{pmatrix} -P\underline{C}+\Gamma Q\Gamma& P\hat{B}\\ \hat{B}^{*}P & -Q \end{pmatrix} < 0, $$

*where* $\hat{A}=(\hat{a}_{ij})_{n\times n}$, $\hat{B}=(\hat {b}_{ij})_{n\times n}$, $\hat{a}_{ij}=\max\{|\underline{a}_{ij}|,|\overline{a}_{ij}|\}$, *and* $\hat{b}_{ij}=\max\{|\underline{b}_{ij}|,|\overline{b}_{ij}|\}$.

### Proof

By Lemma 2, it follows from the LMI (17) that the following condition holds:

19 $$ -\underline{C}P+\Gamma\hat{A}^{*}\hat{A}\Gamma+PP < 0. $$

By the LMI (18), according to Lemma 2, the following condition holds:

20 $$ -P\underline{C}+\Gamma Q\Gamma+P\hat{B}Q^{-1} \hat{B}^{*}P< 0. $$

Summing (19) and (20), we have the following matrix inequality:

21 $$ -\underline{C}P-P\underline{C}+PP+\Gamma\hat{A}^{*}\hat{A}\Gamma+P \hat {B}Q^{-1}\hat{B}^{*}P+\Gamma Q\Gamma< 0. $$

Under the conditions of Theorem 1, system (2) has a unique equilibrium point *ž*. For convenience, we shift the equilibrium to the origin by letting $\tilde{z}(t)=z(t)-\check{z}$, and then system (2) can be transformed into

22 $$ \textstyle\begin{cases} \dot{\tilde{z}}(t)=-C \tilde{z}(t)+A g(\tilde{z}(t))+B g(\tilde {z}(t-\tau)),\\ \Delta\tilde{z}(t)= \tilde{I}(\tilde{z}(t_{k}^{-})), \quad k=1,2,\ldots, \end{cases} $$

where $g(\tilde{z}(t))=f(z(t))-f(\check{z})$ and $\tilde{I}(\tilde {z}(t_{k}^{-}))=-\delta_{ik}\tilde{z}_{i}(t_{k}^{-})$. Meanwhile, the initial condition (4) can be transformed into

23 $$ \tilde{z}(s)=\tilde{\varphi}(s),\quad s\in[-\rho,0], $$

where $\tilde{\varphi}(s)=\varphi(s)-\check{z}\in C([-\rho,0],\mathbb{C}^{n})$.

Consider the following Lyapunov-Krasovskii functional candidate:

24 $$ V \bigl(\tilde{z}(t) \bigr)=V_{1} \bigl(\tilde{z}(t) \bigr)+V_{2} \bigl(\tilde{z}(t) \bigr), $$

where

25 $$\begin{aligned}& V_{1} \bigl(\tilde{z}(t) \bigr)= \sum _{j=1}^{n} p_{j} \tilde{z}^{*}_{j}(t) \tilde {z}_{j}(t), \end{aligned}$$

26 $$\begin{aligned}& V_{2} \bigl(\tilde{z}(t) \bigr)= \sum _{j=1}^{n} q_{j} \int_{t-\tau_{j}}^{t} g^{*}_{j} \bigl(\tilde {z}_{j}(t) \bigr)g_{j} \bigl(\tilde{z}_{j}(t) \bigr) \,\mathrm{d}t. \end{aligned}$$

When $t\neq t_{k}$, $k=1,2,\ldots$ , calculating the upper right derivative of *V* along the solution of (22), applying Lemmas 1 and 4, we get

27 $$\begin{aligned} D^{+}V_{1} \bigl(\tilde{z}(t) \bigr) = & \dot{\tilde{z}}^{*}(t)P \tilde{z}(t)+\tilde {z}^{*}(t)P\dot{\tilde{z}}(t) \\ =& -\tilde{z}^{*}(t)CP\tilde{z}(t)-\tilde{z}^{*}(t)PC \tilde{z}(t)+g^{*} \bigl(\tilde {z}(t) \bigr)A^{*}P\tilde{z}(t) \\ &{}+\tilde{z}^{*}(t)PAg \bigl(\tilde{z}(t) \bigr)+g^{*} \bigl(\tilde{z}(t-\tau) \bigr)B^{*}P\tilde {z}(t)+\tilde{z}^{*}(t)PBg \bigl(\tilde{z}(t-\tau) \bigr) \\ \leq & -\tilde{z}^{*}(t) (CP+PC)\tilde{z}(t)+g^{*} \bigl(\tilde{z}(t) \bigr)A^{*}Ag \bigl(\tilde {z}(t) \bigr)+\tilde{z}^{*}(t)PP\tilde{z}(t) \\ &{}+g^{*} \bigl(\tilde{z}(t-\tau) \bigr)Q g \bigl(\tilde{z}(t-\tau) \bigr)+\tilde {z}^{*}(t)PBQ^{-1}B^{*}P\tilde{z}(t) \\ \leq & \bigl\vert \tilde{z}(t) \bigr\vert ^{*} \bigl(-CP-PC+PP+\Gamma \hat{A}^{*}\hat{A}\Gamma +PBQ^{-1}B^{*}P \bigr) \bigl\vert \tilde{z}(t) \bigr\vert \\ &{}+g^{*} \bigl(\tilde{z}(t-\tau) \bigr)Q g \bigl(\tilde{z}(t-\tau) \bigr) \\ \leq & \bigl\vert \tilde{z}(t) \bigr\vert ^{*} \bigl(-\underline{C}P-P \underline{C}+PP+\Gamma\hat {A}^{*}\hat{A}\Gamma+P\hat{B}Q^{-1}\hat{B}^{*}P \bigr) \bigl\vert \tilde{z}(t) \bigr\vert \\ &{}+g^{*} \bigl(\tilde{z}(t-\tau) \bigr)Q g \bigl(\tilde{z}(t- \tau) \bigr), \end{aligned}$$

28 $$\begin{aligned} D^{+}V_{2} \bigl(\tilde{z}(t) \bigr) =& g^{*} \bigl( \tilde{z}(t) \bigr)Qg \bigl(\tilde{z}(t) \bigr)-g^{*} \bigl(\tilde {z}(t-\tau) \bigr)Qg \bigl(\tilde{z}(t-\tau) \bigr) \\ \leq & \bigl\vert \tilde{z}(t) \bigr\vert ^{*}\Gamma Q\Gamma \bigl\vert \tilde{z}(t) \bigr\vert -g^{*} \bigl(\tilde{z}(t-\tau ) \bigr)Qg \bigl( \tilde{z}(t- \tau) \bigr). \end{aligned}$$

Combining (27) and (28), by (21) we deduce that

29 $$ D^{+}V \bigl(\tilde{z}(t) \bigr)\leq \bigl\vert \tilde{z}(t) \bigr\vert ^{*} \bigl(-\underline{C}P-P\underline {C}+PP+\Gamma\hat{A}^{*}\hat{A} \Gamma+P \hat{B}Q^{-1}\hat{B}^{*}P+\Gamma Q\Gamma \bigr) \bigl\vert \tilde{z}(t) \bigr\vert \leq0. $$

When $t=t_{k}$, $k=1,2,\ldots$ , it should be noted that $V_{2}(t_{k})=V_{2}(t_{k}^{-})$. Then we compute

30 $$\begin{aligned} V \bigl(\tilde{z}(t_{k}) \bigr)-V \bigl(\tilde{z} \bigl(t_{k}^{-} \bigr) \bigr) = & \sum_{j=1}^{n} p_{j} \tilde{z}^{*}_{j}(t_{k}) \tilde{z}_{j}(t_{k})-\sum_{j=1}^{n} p_{j} \tilde{z}^{*}_{j} \bigl(t_{k}^{-} \bigr) \tilde{z}_{j} \bigl(t_{k}^{-} \bigr) \\ =& \sum_{j=1}^{n} (1-\delta_{jk})^{2} p_{j} \tilde{z}^{*}_{j} \bigl(t_{k}^{-} \bigr)\tilde {z}_{j} \bigl(t_{k}^{-} \bigr)-\sum _{j=1}^{n} p_{j} \tilde{z}^{*}_{j} \bigl(t_{k}^{-} \bigr)\tilde{z}_{j} \bigl(t_{k}^{-} \bigr) \\ \leq& 0. \end{aligned}$$

It follows from (29) and (30) that $V(t)$ is non-increasing for $t\geq0$. Then, by the definition of $V(t)$, we infer

31 $$\begin{aligned} V(t) \leq & V(0)=\sum_{j=1}^{n} p_{j} \tilde{z}^{*}_{j}(0)\tilde{z}_{j}(0)+\sum _{j=1}^{n} q_{j} \int_{-\tau_{j}}^{0} g^{*}_{j} \bigl( \tilde{z}_{j}(t) \bigr)g_{j} \bigl(\tilde{z}_{j}(t) \bigr) \,\mathrm{d}t \\ \leq& \sum_{j=1}^{n} p_{j} \bigl\vert \tilde{\varphi}_{j}(0) \bigr\vert ^{2}+\sum _{j=1}^{n} q_{j}\gamma _{j}^{2} \int_{-\tau_{j}}^{0} \bigl\vert \tilde{ \varphi}_{j}(t) \bigr\vert ^{2} \,\mathrm{d}t \\ \leq & \sum_{j=1}^{n} \bigl(p_{j}+\rho q_{j}\gamma_{j}^{2} \bigr)\sup_{t\in[-\rho,0]}\sum_{j=1}^{n} \bigl\vert \tilde{\varphi}_{j}(t) \bigr\vert ^{2} \\ =& \sum_{j=1}^{n} \bigl(p_{j}+ \rho q_{j}\gamma_{j}^{2} \bigr) \bigl\Vert \tilde{\varphi}(t) \bigr\Vert ^{2}. \end{aligned}$$

 □

On the other hand, by the definition of $V(t)$, we have

32 $$ V(t)\geq V_{1}(t)\geq\sum _{j=1}^{n} p_{j} \bigl\Vert \tilde{z}(t) \bigr\Vert ^{2}, \quad t \geq0. $$

From (31) and (32), we obtain

$$\bigl\Vert \tilde{z}(t) \bigr\Vert \leq\sqrt{\frac{\sum_{j=1}^{n} (p_{j}+\tau q_{j}\gamma _{j}^{2})}{\sum_{j=1}^{n} p_{j}}} \bigl\Vert \tilde{\varphi}(t) \bigr\Vert , $$

from which it can be concluded that the origin of (22), or equivalently the equilibrium point of system (1), is globally asymptotically robust stable by the standard Lyapunov theorem. The proof is completed.

If the impulsive operator $I(\cdot)\equiv0$ in (2), we get the following CVNN without impulses:

33 $$ \dot{z}(t)=-C z(t)+A f \bigl(z(t) \bigr)+B f \bigl(z(t-\tau) \bigr)+J, $$

where *C*, *A*, *B*, *J*, and $f(\cdot)$ are defined the same as in (2). Following Theorem 2, we obtain the following corollary on the global robust stability conditions of (33).

### Corollary 1

*Under the conditions of Theorem* 1, *the equilibrium point of system* (33) *is globally asymptotically robust stable*, *if there exist two real positive diagonal matrices* $P=\operatorname{diag}(p_{1},p_{2},\ldots,p_{n})$ *and* $Q=\operatorname{diag}(q_{1},q_{2},\ldots,q_{n})$, *such that the following linear matrix inequalities hold*:

$$ \begin{pmatrix} -\underline{C}P+\Gamma\hat{A}^{*}\hat{A}\Gamma& P\\ P & -I \end{pmatrix} < 0 $$

*and*

$$ \begin{pmatrix} -P\underline{C}+\Gamma Q\Gamma& P\hat{B}\\ \hat{B}^{*}P & -Q \end{pmatrix} < 0, $$

*where* $\hat{A}=(\hat{a}_{ij})_{n\times n}$, $\hat{B}=(\hat {b}_{ij})_{n\times n}$, $\hat{a}_{ij}=\max\{|\underline{a}_{ij}|,|\overline{a}_{ij}|\}$, *and* $\hat{b}_{ij}=\max\{|\underline{b}_{ij}|,|\overline{b}_{ij}|\}$.

### Remark 1

In [9, 13], some dynamic characteristics, such as exponential stability and exponential anti-synchronization, were investigated for real-valued neural networks. Compared to [9, 13], the neural networks model in this paper is complex-valued, which can be viewed as an extension of real-valued neural networks.

### Remark 2

In [33, 34], the criteria for the stability of CVNNs are expressed in terms of complex-valued LMIs. As pointed out in [33], complex-valued LMIs cannot be solved by the MATLAB LMI Toolbox straightforwardly. A feasible approach is to convert complex-valued LMIs to real-valued ones but this could double the dimension of the LMIs. In this paper, we express the stability criteria for CVNNs directly in terms of real-valued LMIs, which can be solved by the MATLAB LMI Toolbox straightforwardly.

### Remark 3

In [2], the authors investigated the problem of global robust stability of recurrent CVNNs with time delays and uncertainties. In Theorem 3.4 of [2], to check robust stability of CVNNs, the boundedness of activation function $f_{i}$ is required. However, in this paper, the boundedness condition is removed. In Example 1, in the next section, the activation function $f_{i}$ is unbounded.

## A numerical example

The following example demonstrates the effectiveness and superiority of our results.

### Example 1

Assume that the network parameters of system (1) are given as follows:

$$\begin{aligned}& \underline{C}= \begin{pmatrix} 0.3 & 0 \\ 0 & 0.3 \end{pmatrix} ,\qquad \underline{A}= \begin{pmatrix} -0.32-0.24\mathrm {i}& -0.18-0.24\mathrm {i}\\ -0.24-0.18\mathrm {i}& -0.24-0.32\mathrm {i}\end{pmatrix} , \\& \overline{A}= \begin{pmatrix} 0.32+0.24\mathrm {i}& 0.18+0.24\mathrm {i}\\ 0.24+0.18\mathrm {i}& 0.24+0.32\mathrm {i}\end{pmatrix} ,\qquad \underline{B}= \begin{pmatrix} -0.24 - 0.18\mathrm {i}& -0.16 + 0.12\mathrm {i}\\ -0.18 - 0.24\mathrm {i}& -0.24 - 0.18\mathrm {i}\end{pmatrix} , \\& \overline{B}= \begin{pmatrix} 0.24 + 0.18\mathrm {i}& 0.12 + 0.16\mathrm {i}\\ 0.18 + 0.24\mathrm {i}& 0.24 + 0.18\mathrm {i}\end{pmatrix} ,\qquad \Gamma= \begin{pmatrix} 0.2 & 0 \\ 0 & 0.2 \end{pmatrix} , \end{aligned}$$

and the impulsive operator $I(\cdot)$ satisfies assumption (**H3**).

Using the above matrices $\underline{A}$, *A̅*, $\underline {B}$, and *B̅*, we have

$$\begin{aligned}& \hat{A}= \begin{pmatrix} 0.4 & 0.3 \\ 0.3 & 0.4 \end{pmatrix} ,\qquad \hat{B}= \begin{pmatrix} 0.3 & 0.2 \\ 0.3 & 0.3 \end{pmatrix}. \end{aligned}$$

Then using YALMIP with the solver of LMILAB, the LMI (10) in Theorem 1, and the LMIs (17) and (18) in Theorem 2, we have the following feasible solutions:

$$\begin{aligned}& U= \begin{pmatrix} 3.7733 & 0 \\ 0 & 3.3139 \end{pmatrix} ,\qquad V= \begin{pmatrix} 3.7733 & 0 \\ 0 & 3.3139 \end{pmatrix} , \\& P= \begin{pmatrix} 0.1854 & 0 \\ 0 & 0.1833 \end{pmatrix} ,\qquad Q= \begin{pmatrix} 0.8824 & 0 \\ 0 & 0.8199 \end{pmatrix}. \end{aligned}$$

Thus, the conditions of Theorems 1 and 2 are satisfied, and system (1) has a unique equilibrium point which is globally asymptotically robust stable. To simulate the results, let us choose *C*, *A*, and *B* from the proper intervals above, and obtain the following specific system:

34 $$ \textstyle\begin{cases} \begin{pmatrix} \dot{z}_{1}(t)\\ \dot{z}_{2}(t) \end{pmatrix} =- \begin{pmatrix} 0.3 & 0\\ 0 & 0.3 \end{pmatrix} \begin{pmatrix} z_{1}(t)\\ z_{2}(t) \end{pmatrix} + \begin{pmatrix} 0.3-0.2\mathrm {i}& 0.15+0.2\mathrm {i}\\ 0.2-0.1\mathrm {i}& 0.2+0.3\mathrm {i}\end{pmatrix} \begin{pmatrix} f_{1}(z_{1}(t))\\ f_{2}(z_{2}(t)) \end{pmatrix} \\ \phantom{ \begin{pmatrix} \dot{z}_{1}(t)\\ \dot{z}_{2}(t) \end{pmatrix} =}{}+ \begin{pmatrix} 0.2+0.15\mathrm {i}& 0.1+0.15\mathrm {i}\\ 0.18-0.24\mathrm {i}& -0.2+0.15\mathrm {i}\end{pmatrix} \begin{pmatrix} f_{1}(z_{1}(t-\tau_{1}))\\ f_{2}(z_{2}(t-\tau_{2})) \end{pmatrix} \\ \phantom{ \begin{pmatrix} \dot{z}_{1}(t)\\ \dot{z}_{2}(t) \end{pmatrix} =}{}+ \begin{pmatrix}0.1-02\mathrm {i}\\ 0.2-0.1\mathrm {i}\end{pmatrix} , \quad t\neq t_{k},\\ \begin{pmatrix} \Delta z_{1}(t_{k})\\ \Delta z_{2}(t_{k}) \end{pmatrix} = \begin{pmatrix} -\delta_{1k} [z_{1}(t_{k}^{-})-(0.4170 - 1.1278\mathrm {i}) ]\\ -\delta _{2k} [ z_{1}(t_{k}^{-})-(0.4863 - 0.4654\mathrm {i}) ] \end{pmatrix}, \quad t=t_{k}, k=1,2,\ldots, \end{cases} $$

where $f_{1}(u)=f_{2}(u)=0.2(e^{u}-1)$, $\delta_{1k}=1+\frac{1}{2}\sin(1+k)$, $\delta_{2k}=1+\frac{2}{3}\cos (2k^{3})$, $k=1,2,\ldots$ , and $t_{1}=0.5$, $t_{k}=t_{k-1}+0.2k$, $k=2,3,\ldots$ .

**Figure 1:**
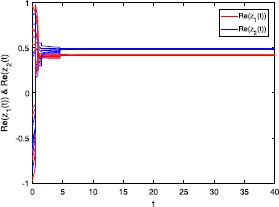
**Real part of the state trajectories for system** (**34**) **with** $\pmb{\tau_{1}=\tau_{2}=0.5}$ **.**

**Figure 2:**
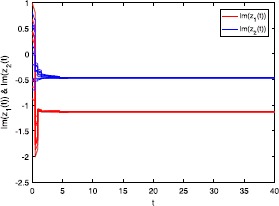
**Imaginary part of the state trajectories for system** (**34**) **with** $\pmb{\tau_{1}=\tau_{2}=0.5}$ **.**

Figures 1 and 2 depict the real and imaginary parts of states of the considered system (34) with $\tau_{1}=\tau_{2}=0.5$, where the initial conditions are with 10 random initial complex-valued points.

**Figure 3:**
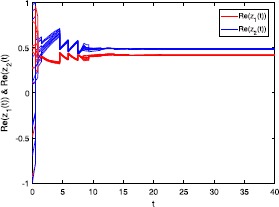
**Real part of the state trajectories for system** (**34**) **with** $\pmb{\tau_{1}=\tau_{2}=8}$ **.**

**Figure 4:**
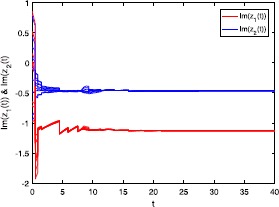
**Imaginary part of the state trajectories for system** (**34**) **with** $\pmb{\tau_{1}=\tau_{2}=8}$ **.**

Figures 3 and 4 depict the real and imaginary parts of states of the considered system (34) with $\tau_{1}=\tau_{2}=8$, where the initial conditions are with 10 random initial complex-valued points.

### Remark 4

In Figures 1-4, we see that the equilibrium point of system (34) is asymptotically stable, whether the delay $\tau_{1}=\tau_{2}=0.5$ or $\tau_{1}=\tau_{2}=8$. It should be noted that the criteria (10), (17), and (18) in Theorems 1 and 2 are independent from the delays *τ*. Therefore, in system (34), the delays have no influence on the stability of the equilibrium point.

## Conclusion

In this paper, we have investigated the existence and uniqueness of the equilibrium as well as its robust stability for an impulsive CVNN with discrete time delays, by applying the homeomorphic mapping theorem and some important inequalities in the complex domain. We have presented some sufficient conditions to guarantee the existence of a unique equilibrium point for the CVNN. In addition, by constructing appropriate Lyapunov-Krasovskii functionals and employing complex-valued matrix inequalities, we also have obtained sufficient conditions to guarantee the robust stability of the CVNN. Finally, a numerical simulation has illustrated the correctness of the proposed theoretical results. Moreover, the conditions in Theorems 1 and 2 are irrelevant to the parameter *τ*, which illustrates that the parameter *τ* has no effect on the uniqueness and existence, neither on the robust stability of system (1). The figures in the article confirm this result.
